# Microwave-Assisted Silanization of Magnetite Nanoparticles Pre-Synthesized by a 3D Microfluidic Platform

**DOI:** 10.3390/nano13202795

**Published:** 2023-10-20

**Authors:** Adelina-Gabriela Niculescu, Alina Moroșan, Alexandra Cătălina Bîrcă, Oana Gherasim, Ovidiu Cristian Oprea, Bogdan Ștefan Vasile, Bogdan Purcăreanu, Dan Eduard Mihaiescu, Marius Rădulescu, Alexandru Mihai Grumezescu

**Affiliations:** 1 Department of Science and Engineering of Oxide Materials and Nanomaterials, Politehnica University of Bucharest, 011061 Bucharest, Romania; adelina.niculescu@upb.ro (A.-G.N.); alexandra.birca@upb.ro (A.C.B.); bogdan.vasile@upb.ro (B.Ș.V.); bogdanpb89@gmail.com (B.P.); grumezescu@yahoo.com (A.M.G.); 2 Research Institute of the University of Bucharest—ICUB, University of Bucharest, 050657 Bucharest, Romania; 3 Department of Organic Chemistry, Politehnica University of Bucharest, 011061 Bucharest, Romania; alina.morosan@upb.ro; 4 Lasers Department, National Institute for Lasers, Plasma and Radiation Physics, 409 Atomistilor St., 077125 Magurele, Romania; oana.gherasim@inflpr.ro; 5 Department of Inorganic Chemistry, Physical Chemistry and Electrochemistry, University Politehnica of Bucharest, 1-7 Polizu St., 011061 Bucharest, Romania; ovidiu73@yahoo.com (O.C.O.); radulescu_marius@yahoo.com (M.R.); 6 BIOTEHNOS S.A., Gorunului Rue, No. 3-5, 075100 Otopeni, Romania; 7 Academy of Romanian Scientists, Ilfov No. 3, 050044 Bucharest, Romania

**Keywords:** iron oxide synthesis, magnetite nanoparticles, microfluidic synthesis, 3D microfluidic platform, microwave-assisted functionalization

## Abstract

Magnetite nanoparticles (Fe_3_O_4_ NPs) are among the most investigated nanomaterials, being recognized for their biocompatibility, versatility, and strong magnetic properties. Given that their applicability depends on their dimensions, crystal morphology, and surface chemistry, Fe_3_O_4_ NPs must be synthesized in a controlled, simple, and reproducible manner. Since conventional methods often lack tight control over reaction parameters and produce materials with unreliable characteristics, increased scientific interest has been directed to microfluidic techniques. In this context, the present paper describes the development of an innovative 3D microfluidic platform suitable for synthesizing uniform Fe_3_O_4_ NPs with fine-tuned properties. On-chip co-precipitation was performed, followed by microwave-assisted silanization. The obtained nanoparticles were characterized from the compositional and microstructural perspectives by X-ray diffraction (XRD) and transmission electron microscopy (TEM). Moreover, supplementary physicochemical investigations, such as Fourier Transform Infrared Spectroscopy (FT-IR), Kaiser Test, Ultraviolet-Visible (UV-Vis) Spectrophotometry, Dynamic Light Scattering (DLS), and Thermogravimetry and Differential Scanning Calorimetry (TG-DSC) analyses, demonstrated the successful surface modification. Considering the positive results, the presented synthesis and functionalization method represents a fast, reliable, and effective alternative for producing tailored magnetic nanoparticles.

## 1. Introduction

Nanotechnology is gaining increasing popularity for advancing various sides of science as it allows matter manipulation on a scale where materials display different features than micro-/macro-scale counterparts. The unique, appealing physicochemical properties (correlated to nanomaterials’ specific sizes and morphologies, chemical composition, charge, crystalline structure, and solubility) render nanodimensional materials suitable for numerous and broad applications [1,2,3,4,5,6].

Among all investigated nanomaterials, magnetic nanoparticles (NPs) have received the most interest, given their attractive characteristics. These materials are especially recognized for their biocompatibility, chemical stability, low price, and size-dependent magnetic activity [7,8]. From the class of magnetic nanoparticles, magnetite (Fe_3_O_4_) has been particularly remarked, as it shows the strongest magnetism compared to other transition metal oxides [9] while also benefiting from availability, versatility, and eco-friendliness [10]. Fe_3_O_4_ NPs possess superparamagnetism, high saturation field, and high magnetic susceptibility, with their distinctive features being attributed to the transfer of ions from Fe^2+^ to Fe^3+^ [1,11]. These valuable magnetic properties rendered Fe_3_O_4_ NPs suitable for a plethora of utilizations, including bio-sensing and diagnosis [12,13,14], contrast agents [13,14,15,16,17,18], cancer treatment [13,14,18,19,20,21,22,23,24], hyperthermia therapy [13,14,15,20,21,25], controlled and targeted drug delivery [13,18,19,22,26,27,28,29,30], catalysis [31,32,33], batteries [34,35,36], magnetic inks [37,38,39], data storage [40,41,42], and water decontamination [43,44,45,46,47].

Despite being relatively stable at room temperature, Fe_3_O_4_ NPs tend to oxidize, quickly transforming into maghemite [9]. Moreover, pristine magnetite NPs also exhibit an agglomeration tendency. To avoid these undesired effects, particles are usually functionalized or surface-coated by various compounds, such as polymers, metals, or organic and/or inorganic stabilizing agents [10]. Magnetic NPs with dimensions below 100 nm display favorable surface reactivity, enabling easy ligand attachment and small settling velocities that correlate with high suspension stability. In addition, the iron atoms from the surface of the particles that are not bound to oxygen atoms coordinate with water molecules that dissociate and lead to the production of Fe-OH groups. These preformed hydroxyls exhibit an amphoteric character, being able to further react as either bases or acids [9].

As the behavior of Fe_3_O_4_ NPs is deeply related to their dimensions, crystal morphology, and surface chemistry, the synthesis process requires simplicity, reproducibility, and repeatability [10,48,49]. The most used and most efficient synthesis method is the chemical co-precipitation of iron salts with a base [9,50,51]. Despite being a simple and low-cost method for producing hydrophilic particles, co-precipitation offers limited control over size, size distribution, crystallinity, and magnetic properties during synthesis and generates batch-to-batch variations [50,51,52]. Alternative methods such as micro-emulsion technique and thermal decomposition of organometallic precursors have been noted to provide better tuning of the particles’ morphology, size, and monodispersion. Nonetheless, they generally employ expensive toxic chemicals, high pressure and temperature, and long synthesis times [50,52].

Thus, when Fe_3_O_4_ NPs with specified characteristics and tailored properties must be obtained, conventional synthesis methods do not provide tight control over experimental variables, generating particles with a broad size distribution, large inter-batch variability, irregular structures, and unreliable properties [53,54,55,56]. Additionally, classic syntheses may affect the environment, representing pollutant sources and high-energy consumers. Moreover, traditional processes necessitate large spaces and expensive equipment, high operating costs, complex stepwise procedures, insufficient control over mixing, poor reproducibility, long reaction times, and safety concerns [51,57,58].

To overcome the challenges of conventional syntheses, microfluidic technology has emerged as a promising solution. By fluid manipulation in microscale channels and chambers, microfluidic devices are excellent synthesis platforms for NPs with controlled properties and functions [2,59,60,61]. Enabling a remarkable control over chemical substances’ spatial and temporal distribution, microfluidic platforms yield nanomaterials with precise dimensions, narrow size distribution, uniform shape, and engineered surface composition [54,59,61,62]. Moreover, microfluidic synthesis methods outperform large-scale systems, demonstrating high process reproducibility, defined mixing, fast heat and mass transfer, rapid chemical reactions, ease of automation, and high throughput [7,50,60,61,62,63].

Given their appealing features, microfluidic devices started being involved in numerous syntheses, providing a rapid, low-cost, controllable, sustainable, and reliable method for fabricating a wide range of nanomaterials, including magnetite [61,63]. However, during Fe_3_O_4_ NPs synthesis, microreactor channels can get clogged due to the high reactivity of iron precursor and the large surface-to-volume ratio of the products [50]. Other issues contributing to particle precipitation and fouling microscale channels within typical platforms include inadequate mixing over short channel lengths and wide ranges of Reynolds numbers [51,64]. The most simple manner to improve mixing efficiency and avoid channel clogging is to conveniently alter channel geometry [61]. In particular, moving from common planner geometrical patterns to 3D micromixers holds great promise for expanding the potential of microfluidic techniques. Adding a third dimension in fluid manipulation increases fluid contact times, surface disruption, and channel length reduction, all contributing to enhancing mixing efficiency. However, 3D micromixers are a developing technology that has not reached industrial translation yet, mainly due to their more difficult fabrication and delicate parameter optimization [61,64].

In this context, this study has focused on developing an innovative 3D microfluidic platform suitable for Fe_3_O_4_ NPs synthesis. Specifically, a novel microfluidic device was designed, fabricated, and tested for magnetite production. To avoid undesired surface oxidation and particle agglomeration, the microfluidic-obtained NPs were further functionalized with (3-aminopropyl) triethoxysilane (APTES) through a microwave-assisted method. In addition, Fe_3_O_4_ NPs were thoroughly evaluated by a series of physicochemical characterization methods: X-ray diffraction (XRD), Transmission Electron Microscopy (TEM), Selected Area Electron Diffraction (SAED), Fourier Transform Infrared Spectroscopy (FT-IR), Kaiser Test, Ultraviolet-Visible (UV-Vis) Spectrophotometry, Dynamic Light Scattering (DLS), and Thermogravimetry and Differential Scanning Calorimetry (TG-DSC).

## 2. Materials and Methods

### 2.1. Materials

Ferric chloride (FeCl_3_), iron sulfate heptahydrate (FeSO_4_·7H_2_O), (3-aminopropyl) triethoxysilane (APTES), potassium cyanide (KCN), and phenol were purchased from Sigma Aldrich Merck (Darmstadt, Germany); sodium hydroxide (NaOH) was purchased from Lach-Ner (Tovarni, Czech Republic); ethanol and acetic acid were purchased from Emsure Merck Millipore (Darmstadt, Germany); pyridine and ninhydrin were purchased from Merck (Darmstadt, Germany). All the reagents utilized in this study were of analytical purity and used as received. Ultrapure water was used for all experiments.

### 2.2. Microfluidic Platform Fabrication

**Figure 1:**
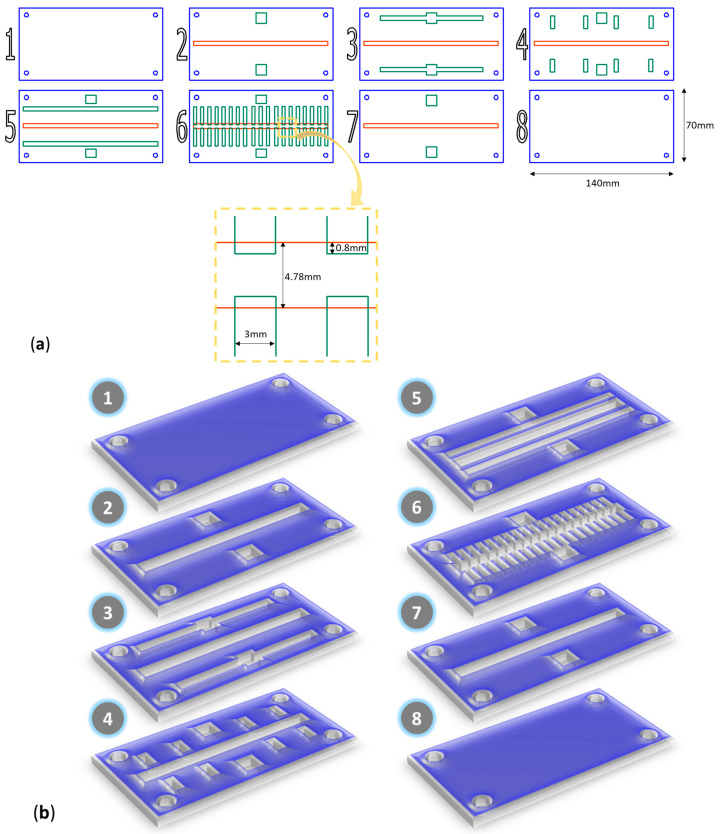
Microfluidic platform configuration. (**a**) Proportional 2D schematic representation of the platform layers. (**b**) 3D schematic representation of the platform layers.

The 3D microfluidic synthesis platform design was created using RDWorksV8 software dedicated to laser cutting machine equipment. The micromixer comprises 8 layers of the same dimensions (i.e., width-length = 140 mm-70 mm), with the patterns indicated in Figure 1. The model was fabricated with the aid of the 1610 Pro laser cutting machine (RUBIQ CNC, Bacău, Romania) on 2 mm-thick PMMA sheets. The layers were aligned and tightened together by 4 screws (4 mm in diameter), and the margins were sealed with a commercial bicomponent epoxy adhesive (“Epoxy Universal”, Bison International B.V., Goes, The Netherlands).

### 2.3. Nanoparticle Preparation

The precursor solution was prepared by dissolving FeCl_3_ and FeSO_4_·7H_2_O in a 1 to 6 weight ratio in 300 mL of ultrapure water, while the precipitating agent solution consisted of 300 mL of 1.25 M NaOH aqueous solution. Iron oxide nanoparticles were obtained through the co-precipitation of the iron ions at the contact point with the alkaline solution. Reagent solutions were simultaneously introduced in the experimental setup using a classical osmosis pump with a 90 mL/s flow rate. In more detail, the iron precursor solution and the precipitating solution were circulated through the channels represented with green in Figure 1, while the central red channel acted as a vertical mixing chamber. Thus, multipoint 3D mixing was achieved within the microfluidic platform, subsequently increasing fluid contact times and improving mixing efficiency.

The obtained Fe_3_O_4_ NPs were separated using a neodymium magnet. Then, they were washed with ultrapure water, dispersed by ultrasonication, and centrifuged for 40 min at 8000 rpm. These processes were repeated three times. After that, the processes were repeated three more times using ethanol (with a trace amount of acetic acid).

The purified NPs were dispersed in ethanol, APTES solution (i.e., 10 mL APTES in 50 mL ethanol) was added under continuous stirring, and the resulting mixture was heated using microwave irradiation for 30 min (performed in a MW4717 (Stuttgart, Germany), rated microwave power output 600 W, microwave frequency 2450 MHz). The final nanostructured products were further subjected to several series of washing with ethanol, ultrasonication, and centrifugation to eliminate potential traces of unreacted compounds.

### 2.4. Characterization Methods

#### 2.4.1. X-ray Diffraction (XRD)

An X-ray diffraction analysis of nanomaterial powders was accomplished with the aid of a Panalytical Empyrean diffractometer (PANalytical, Almelo, The Netherlands) equipped with a CuKα radiation source (λ = 1.056 Å) at 40 mA and 45 kV. Samples were scanned at room temperature, with determinations in the Bragg diffraction angle range between 10° and 80°.

#### 2.4.2. Transmission Electron Microscopy (TEM) and Selected Area Electron Diffraction (SAED)

The sample was dispersed in ethanol through a 15-min ultrasonic treatment. Then, a small amount of it was placed on a carbon-copper grid and dried at room temperature. For TEM micrographs recording, a high-resolution 80–200 Titan Themis transmission electron microscope from ThermoFisher Scientific (Hillsboro, OR, USA) was operated in the transmission mode at a 200 kV voltage, with point and line resolutions of 2 Å and 1 Å, respectively. Additional crystallographic data was acquired using the equipment’s SAED accessory (ThermoFisher Scientific, Hillsboro, OR, USA).

#### 2.4.3. Fourier Transform Infrared Spectroscopy (FT-IR)

The synthesized nanoparticles were characterized using a Nicolet iS50FT-IR (ThermoFisher Scientific, Waltham, MA, USA) spectrometer. The measurements were performed at room temperature in the range of 4000–400 cm^−1^, using the resolution of 8 cm^−1^. All spectra were registered in attenuated total reflectance (ATR) mode using a diamond crystal. OmnicPicta software (version 8.2, Thermo Fischer Scientific, Madison, WI, USA) was used to co-add and process the 96 scans acquired for each sample.

#### 2.4.4. Kaiser Test and Ultraviolet-Visible (UV-Vis) Spectrophotometry

For the Kaiser detection, fresh test solutions were prepared and added to a small amount of sample placed in a test tube, as described in reference [65]. The test tube was further put in a sand bath and kept at a temperature of 105–110 °C for 5 min.

The resulting solutions were further analyzed using an Evolution 300 UV-Vis spectrophotometer (ThermoFisher Scientific, Madison, WI, USA). The absorbance values were measured in standard quartz cuvettes between 400 and 800 nm with a bandwidth of 2.0 nm and a scan speed of 240 nm/min. The acquired data were processed using the VISIONpro dedicated software (version 2.0).

#### 2.4.5. Dynamic Light Scattering (DLS)

The synthesized nanoparticles were dispersed in water, sonicated for 5 min, placed in dedicated cuvettes (DTS0012), and subjected to DLS analysis using a Nano ZS Zetasizer (Malvern Instruments, Malvern, UK). Measurements were performed at a spreading angle of 90° and a temperature of 25 °C; the reported values for average hydrodynamic diameter, polydispersity index, and Zeta potential are the average of five measurements.

#### 2.4.6. Thermogravimetry and Differential Scanning Calorimetry (TG-DSC)

For the realization of the thermal analysis, an STA 449C Jupiter device from Netzsch (NETZSCH-Gerätebau GmbH, Selb, Germany) was employed. A small amount of sample was placed in an open alumina crucible and heated from room temperature up to 900 °C, at a heating rate of 10 °C min^−1^, under a 50 mL min^−1^ dried airflow. As a reference, an empty alumina crucible was used. In addition, the evolved gases were studied by a thermostat gas-cell-equipped FTIR Tensor 27 from Bruker (Bruker Co., Ettlingen, Germany).

## 3. Results

**Figure 2:**
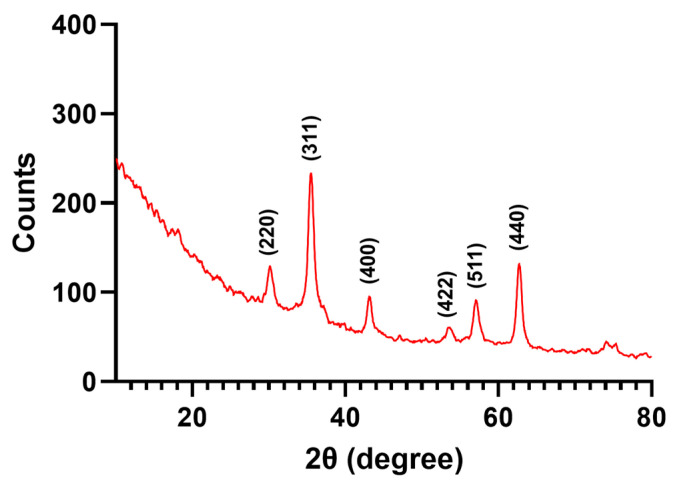
X-ray diffractogram of pristine Fe_3_O_4_ NPs.

The X-ray diffractogram of pristine Fe_3_O_4_ NPs is presented in Figure 2. The identified diffraction peaks correspond to the (220), (311), (400), (422), (511), and (440) diffraction planes of the crystallographic system. According to JCPDS 01-084-2782, these are distinctive for crystalline magnetite with a spinel cubic structure.

**Figure 3:**
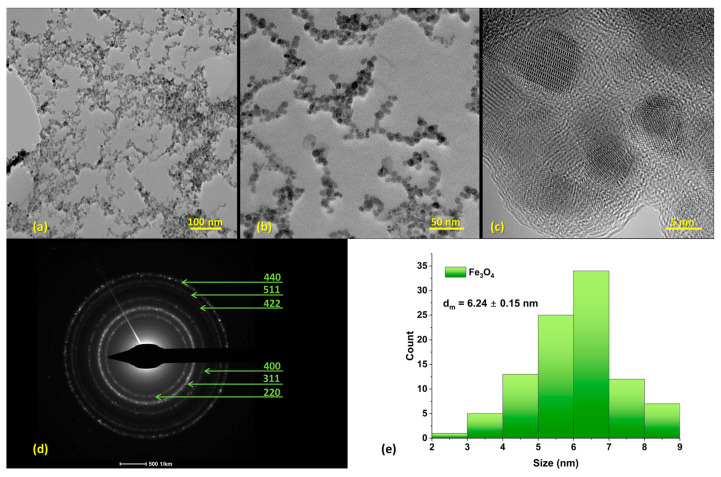
(**a**–**c**) TEM micrographs of pristine Fe_3_O_4_ NPs (Scale bars: (**a**) 100 nm; (**b**) 50 nm); (**c**) 5 nm). (**d**) SAED diffraction pattern with the corresponding Miller indices for the pristine Fe_3_O_4_ NPs. (**e**) Size distribution of nanoparticles.

Further, TEM images evidenced the formation of ultra-small particles (with 6.24 ± 0.15 nm average particle size) with monomodal size distribution (Figure 3e), exclusive spherical morphology (Figure 3b), and reduced aggregation (Figure 3a). This is due to the presence of the outer dispersant layer on the NPs’ surface, which was observed in the HR-TEM micrograph (Figure 3c). Moreover, SAED analysis of the pristine Fe_3_O_4_ NPs (Figure 3d) recorded 6 concentric rings formed at (220), (311), (400), (422), (511), and (440), agreeing with previously obtained XRD data and confirming the crystalline nature of the prepared material.

**Figure 4:**
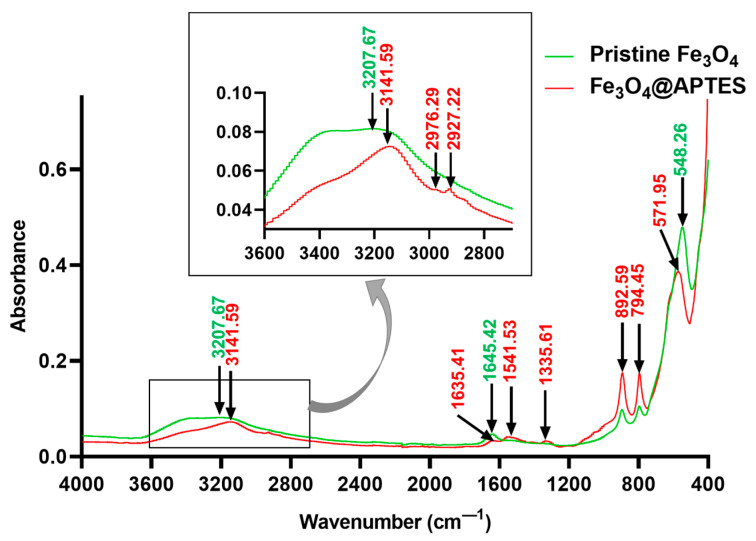
Comparison between MID-IR spectra of pristine Fe_3_O_4_ and Fe_3_O_4_@APTES NPs.

To confirm the successful silanization of the magnetite NPs, FT-IR analysis was performed (Figure 4). The FT-IR spectra of pristine Fe_3_O_4_ and Fe_3_O_4_@APTES NPs both exhibited a wide absorption band around 3400 cm^−1^, attributed to O–H stretching vibration, and a small peak around 1640 cm^−1^, correlated with O–H deformed vibration, which demonstrated the presence of OH groups on the surface of obtained NPs. The absorption bands specific to Fe–O stretching vibrations were also identified in both spectra at 548 cm^−1^ for bare magnetite and 571 cm^−1^ for the functionalized sample. The shift to a higher wavenumber noticed in the Fe_3_O_4_@APTES can be explained by the formation of Fe–O–Si bonds, reflecting the replacement of Fe–O–H groups on the particle surface with Fe–O–Si(O–)_2_–(CH_2_)_3_–NH_2_. As –Si(O–) has a greater electronegativity than H, the forces of Fe–O bonds are enhanced, and the absorbance bands are shifted to higher wavenumber values [66]. The presence of the propyl group from APTES is also proven by the bands at 2976 cm^−1^ and 2927 cm^−1^ attributed to C–H stretching. Additionally, the 1541 cm^−1^ peak corresponds to the N–H bending vibration.

**Figure 5:**
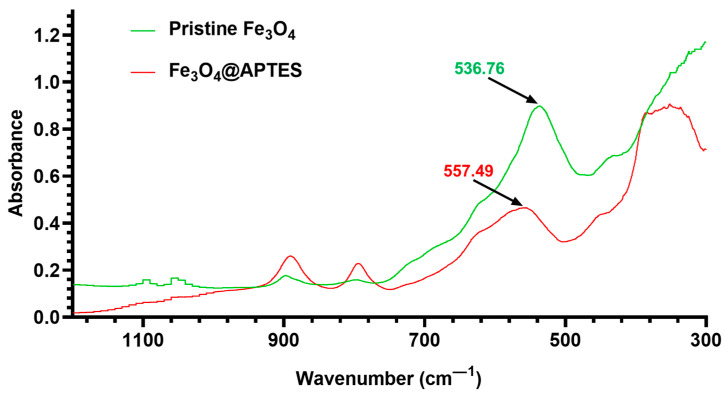
Comparison between FAR-IR spectra of natural Fe_3_O_4_, synthesized pristine Fe_3_O_4_, and Fe_3_O_4_@APTES NPs.

Moreover, FAR-IR spectra (Figure 5) prove a shift of the 536 cm^−1^ peak to 557 cm^−1^, demonstrating a modification of surface interaction forces from Fe–O–H to Fe–O–Si correlated with a force constant modification of the Fe–O bond. There is also a considerable absorption difference between non-functionalized magnetite and APTES-functionalized one, related to the significant mitigation of the Fe–O stretching absorption (557 cm^−1^ shifted peak).

**Figure 6:**
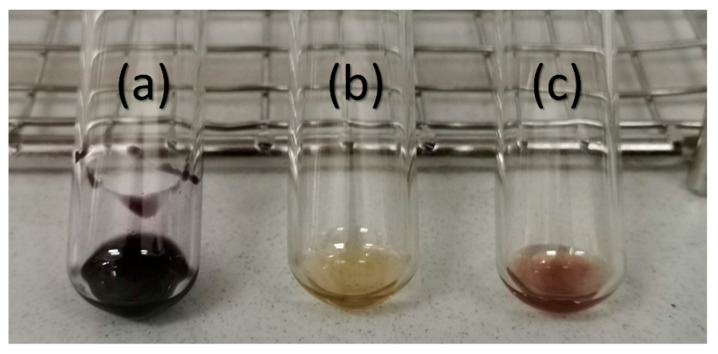
Kaiser test performed on (**a**) APTES, (**b**) pristine Fe_3_O_4_ NPs, (**c**) Fe_3_O_4_@APTES NPs.

The Kaiser test allowed for the qualitative determination of the amino groups’ presence in the surface-modified iron oxide nanoparticles. The colorimetric assay was performed on the functionalization agent (i.e., APTES), pristine Fe_3_O_4_ NPs, and Fe_3_O_4_@APTES NPs, leading to the appearance of a violet shade, no color change, and dark brown shade, respectively (Figure 6).

**Figure 7:**
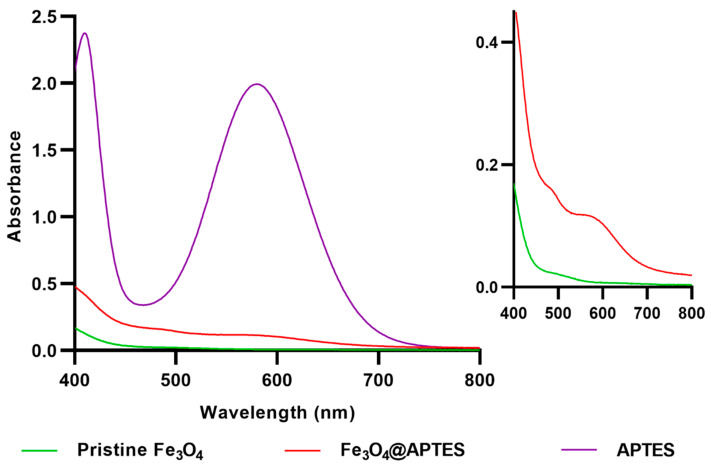
Overlapped display of UV-Vis spectra of ethanol, APTES, pristine Fe_3_O_4_ NPs, and Fe_3_O_4_@APTES NPs.

Despite the noticeable difference in color between the pristine Fe_3_O_4_ NPs and the functionalized ones, the expected blue-violet shade is covered by the color intensity of the dispersed NPs. For better validation of the presence of APTES within the surface-modified sample, UV-Vis analysis was performed on the solutions resulting from the Kaiser test (Figure 7). Thus, it was observed that the absorbance maximum of Fe_3_O_4_@APTES NPs is in the same wavelength range as for the organosilane sample. This demonstrates the formation of the Ruhemann complex [67], which does not appear in the pristine magnetite sample, thus confirming the successful functionalization of the Fe_3_O_4_ NPs.

**Figure 8:**
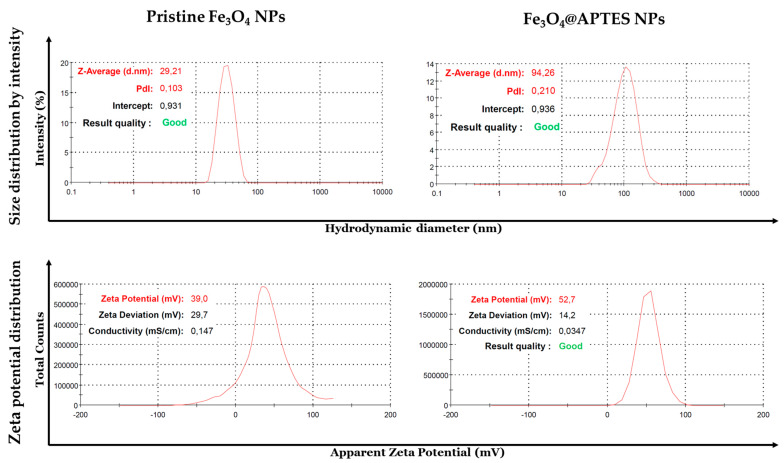
DLS analyses for pristine Fe_3_O_4_ NPs and Fe_3_O_4_@APTES NPs.

DLS analysis allowed the comparison of the fabricated materials in terms of size and colloidal stability (Figure 8). Pristine Fe_3_O_4_ NPs displayed an average hydrodynamic diameter of 29.21 nm, with a polydispersity index of 0.103, indicating good size homogeneity. After functionalization with APTES, the NPs’ hydrodynamic diameter increased by 3 times while maintaining good uniformity (0.210 polydispersity index). Moreover, an increase in the Zeta potential was also observed, changing from 39.0 mV (pristine Fe_3_O_4_ NPs) to 52.7 mV (Fe_3_O_4_@APTES NPs), demonstrating the improved colloidal stability following silanization.

**Figure 9:**
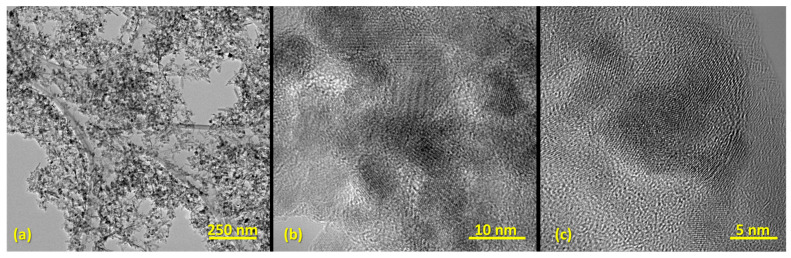
(**a**–**c**) TEM micrographs of Fe_3_O_4_@APTES NPs (Scale bars: (**a**) 250 nm; (**b**) 10 nm); (**c**) 5 nm).

Besides, the increase in size caused by shell addition onto the magnetic cores is visible in TEM micrographs (Figure 9), which also depict the dimensional and morphological uniformity of the functionalized nanomaterials.

**Figure 10:**
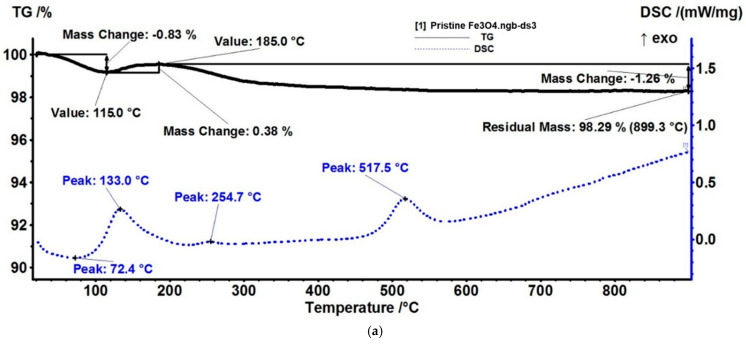

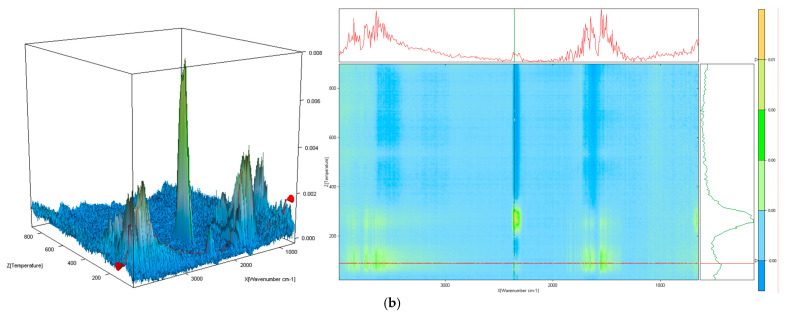
Pristine Fe_3_O_4_-NPs: (**a**) Thermogravimetric analysis, (**b**) FTIR 3D diagram and its 2D projection in temperature/wavenumber plane ((**top**) FTIR spectrum at 87 °C, (**right view**) trace for CO_2_ at 2355 cm^−1^).

The thermogravimetric analysis of the pristine Fe_3_O_4_ NPs shows that the sample loses 0.83% of its initial mass up to 115 °C. The process is accompanied by an endothermic effect on the DSC curve, with a minimum at 72.4 °C, indicating the most probable cause of the elimination of residual water molecules from the nanoparticles’ surface. The FTIR 3D diagram and 2D projection confirm that the evolved gases contain water vapors (Figure 10). Between 115–185 °C, the sample is gaining mass; as Fe(II) is oxidized to Fe(III), the transformation of Fe_3_O_4_ to γ-Fe_2_O_3_ is accompanied by an exothermic effect on the DSC curve, with the maximum at 133 °C [68]. After 185 °C, the sample is slowly losing mass, with the FTIR spectra of evolved gases indicating the presence of water and carbon dioxide at 263 °C, while the DSC curve presents a weak exothermic peak at 254.7 °C. This indicates the oxidation of some organic impurities from the surface of nanoparticles. At 517.5 °C, the DSC curve exhibits an exothermic effect with no mass loss, which indicates a phase transition. This is typical for the transformation of maghemite to hematite [69]. The residual reddish-brown mass represents 98.29%.

**Figure 11:**
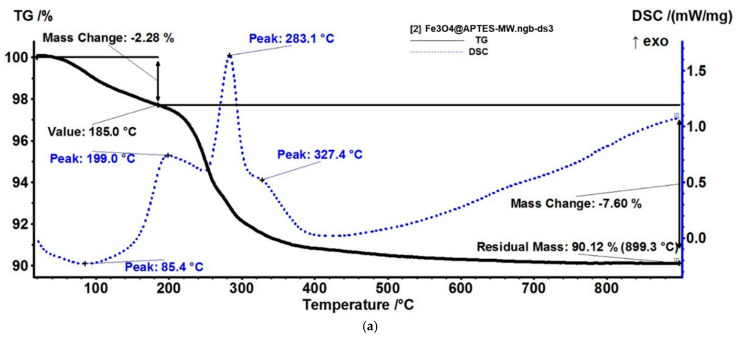

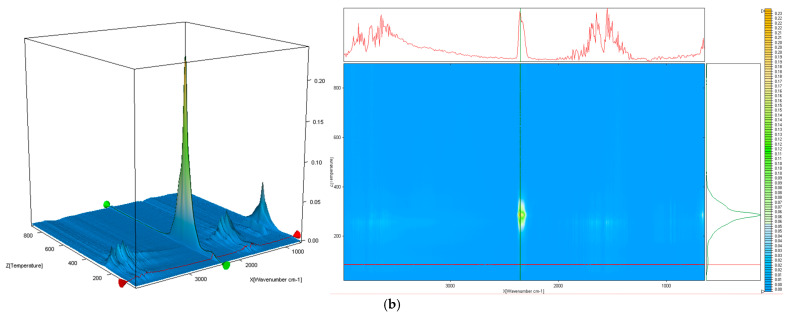
Fe_3_O_4_@APTES-MW NPs: (**a**) Thermogravimetric analysis, (**b**) FTIR 3D diagram and its 2D projection in temperature/wavenumber plane ((**top**) FTIR spectrum at 82 °C, (**right view**) trace for CO_2_ at 2355 cm^−1^).

The Fe_3_O_4_@APTES-MW NPs sample loses 2.28% of its mass up to 185 °C in an endothermic process, with the identified products in the evolved gases being water and carbon dioxide (Figure 11). The mass loss is larger than the simple magnetite sample, indicating that more water molecules remain trapped inside the APTES shell. After 185 °C, the sample suffers the main degradation step, losing 7.60% of its mass. The process is accompanied by a complex, three-peak exothermic effect on the DSC curve (at 199, 283.1, and 327.4 °C). This indicates that the oxidation of the APTES shell occurs in discrete steps, with the main degradation products being H_2_O and CO_2_, while the iron oxide is encased in a silica shell. The slow mass loss recorded between 350–700 °C is due to silica densification by condensation of Si–OH moieties [70]. The absence of the exothermic effect at ~500–600 °C, generally attributed to maghemite’s transformation to hematite, demonstrates that the iron oxide core is protected from thermal oxidation by silica shell [71]. The residual mass represents 90.12%. Based on thermal analysis results, the load of APTES on Fe_3_O_4_ NPs was estimated at ~8.8%.

## 4. Discussion

The newly developed 3D microfluidic platform has allowed for the successful synthesis of ultrasmall magnetic materials in a controlled manner and within a short reaction time (less than 1 min). Performing the co-precipitation on-chip allowed enhanced control over reaction parameters, with the vertical mixing chamber enabling efficient mixing. Compared with existent microfluidic devices for magnetite synthesis [10], this study provides an innovative method for magnetite nanoparticle production, suggesting a 3D mixing approach at high flow rates. In comparison to laminar flow 2D devices, the proposed device is able to increase the nanoparticle generation by several orders of magnitude. Instead of creating a reaction area at the horizontal intersection of 2 reagent channels, this platform allows multipoint 3D mixing of reagents, subsequently improving mixing efficiency and significantly decreasing reaction time. Another advantage of this technique is the dispersibility yield; using the 3D device, we obtained almost quantitative dispersibility (this is not the case for classical approaches). To our knowledge, this is the first time such a 3D multilayered microreactor has been designed, fabricated, and used for magnetite synthesis.

Moreover, microfluidic 3D mixing is an emerging technology, and only a few designs have been reported in the literature [61]. The other three-dimensional configurations available in the literature comprise a 3D co-flow microfluidic device for synthesizing polymer particles [72], a 3D modular microfluidic platform for core-shell droplet generation [73], and a star-shaped 3D structure with multiple inlets for producing smaller droplets than conventional microfluidic devices [74].

Based on thorough characterization (i.e., XRD, TEM, and SAED analyses), it was established that the obtained nanoparticles consisted of crystalline magnetite with spherical morphology. This shape is the most found among microfluidic synthesis methods, with numerous studies reporting on-chip fabricated Fe_3_O_4_-based nanospheres [50,62,75,76,77]. Nonetheless, through the well-controlled variation of operational parameters (e.g., reagents flow, residence time, temperature, channel geometry), other morphologies can be obtained, such as octahedral-shaped nanocrystals [78], hexagonal plates [78], and tadpole-like particles [79].

Magnetic nanoparticles employed in biomedical applications normally have sizes below 20 nm, a suitable dimension for enabling their superparamagnetic behavior [1]. Our study has led to obtaining Fe_3_O_4_ NPs of diameters below 6.5 nm, a dimension that agrees with the requirements for further applications in biomedicine. Moreover, the proposed synthesis device enabled the fabrication of much smaller magnetite particles than our research group obtained with planar microreactors (i.e., 20 to 50 nm) [77]. Other scientists have also reported similar sizes when utilizing typical microfluidic devices, as Bemetz and colleagues obtained NPs of 25 nm [50]. However, Kašpar et al. [62] managed to obtain particles of 4–7 nm utilizing 2D platforms, with these favorable dimensions being attributed to the reduced microchannel diameters (i.e., 20–60 μm). Differently, Suryawanshi et al. [80] have used a continuous flow spiral microreactor that allowed the formation of Fe_3_O_4_ NPs with a mean particle size of less than 10 nm.

Silanes represent commonly used bifunctional modifiers for metal oxide NPs functionalization [81]. We used APTES as a coupling agent to prevent Fe_3_O_4_ NPs agglomeration through steric repulsion. Moreover, its terminal amine group enables further bioconjugation or can act as a linker in synthesizing composite/hybrid structures made of Fe_3_O_4_ NPs and other inorganic materials. Furthermore, the presence of the amino group on the surface of magnetite nanoparticles allows their further modification with other functional groups, including peptides, antibodies, oligonucleotides, or polymers, toward creating vehicles for targeted drug delivery [82]. Considering these advantageous properties of Fe_3_O_4_@APTES NPs, several studies have reported their fabrication [83,84,85,86], as depicted in Table 1.

Compared to literature studies, the proposed fabrication method for Fe_3_O_4_@APTES NPs drastically reduced preparation time, required simpler and fewer steps, and did not employ an argon/nitrogen atmosphere. The described functionalization technique resulted in the successful covering of magnetite NPs with an organosilane shell, as demonstrated by FT-IR, Kaiser, UV-Vis, and TG-DSC characterizations. Thus, the obtained materials can be employed in further applications, including composite synthesis, nanostructured catalytic materials, targeted drug delivery vehicles, wastewater treatment, and nanophase synthesis [82,83,84,85,86,87,88].

## 5. Conclusions

Conventional magnetite synthesis methods often lead to the formation of particles with variable features, whereas the utilization of typical microreactor systems is limited by their clogging tendency. Therefore, this study offers an improved alternative to existing Fe_3_O_4_ NPs fabrication methods, proposing a 3D microfluidic platform able to generate uniform magnetic NPs. Thorough physicochemical investigations (i.e., XRD, TEM, and SAED) revealed that the obtained Fe_3_O_4_ NPs were crystalline, with ultrasmall sizes (below 6.5 nm) and exclusive spherical morphology. To prevent agglomeration, the pristine particles were further surface-modified by microwave-assisted functionalization with an organosilane (i.e., APTES). The successful grafting of the coupling agent was confirmed by a series of characterization methods, including FT-IR, Kaiser, UV-Vis, DLS, TEM, and TG-DSC analyses. The proposed method for the fabrication of Fe_3_O_4_@APTES NPs required less time, fewer steps, and simpler operations than the previously reported techniques in the literature. Thus, it can be concluded that the developed device provides a reliable synthesis platform for the formation of fine-tuned magnetic materials in a fast and effective manner.

## Figures and Tables

**Table 1:** Studies on the fabrication of Fe_3_O_4_—APTES NPs.

Reagents	Synthesis Method	APTES Functionalization Method	Ref.
FeCl_2_ and FeCl_3_ hexahydrate salts Aqueous ammonia solution	Modified Massart co-precipitation method	The reaction was carried out for 24 h at a constant temperature of 50 °C	[83]
FeCl_2_ and FeCl_3_ anhydrous salts dissolved in 0.1 HCl solution 1.5 M NH_3_ solution	Modified co-precipitation method (precursor and precipitant solution were stirred for 2 h at 40 °C)	Fe_3_O_4_ NPs dispersion in ethanol was bubbled with argon gas for 30 min, APTES was added under mechanical stirring, and the mixture was left to react for 24 h at room temperature	[84]
Fe(NO_3_)_3_ and FeSO_4_ heptahydrate NaOH solution	Co-precipitation method (alkaline solution was heated to 85 °C under argon atmosphere, iron precursor solution was added dropwise while stirring vigorously, and the mixture was left to react for 1 h)	APTES was added to Fe_3_O_4_ NPs dispersion in ethanol/water (volume ratio, 1:1) solution and the mixture was stirred under argon atmosphere for 24 h at 40 °C	[85]
FeCl_3_ hexahydrate salt and FeSO_4_ heptahydrate NaOH solution	Modified co-precipitation method (iron precursor solution was stirred at 60 °C for 3 h under nitrogen atmosphere and alkaline solution was added dropwise)	APTES was added to Fe_3_O_4_ NPs suspension under nitrogen atmosphere, and the mixture was left to react under stirring for 24 h at 40 °C	[86]
FeCl_3_ anhydrous salt and FeSO_4_ heptahydrate NaOH solution	Microfluidic-assisted co-precipitation (less than 1 min)	Microwave-assisted reaction carried out for 30 min	This study

## Data Availability

Not applicable.
